# Antenatal education and the birthing experience of Brazilian women: a qualitative study

**DOI:** 10.1186/1471-2393-13-171

**Published:** 2013-09-05

**Authors:** Maria Amelia Miquelutti, José Guilherme Cecatti, Maria Yolanda Makuch

**Affiliations:** 1 Departament of Obstetrics and Gynaecology, School of Medical Sciences, University of Campinas (UNICAMP), Campinas, SP, Brazil; 2 Center for Research on Reproductive Health of Campinas (Cemicamp), Caixa Postal 6181, 13084-971 Campinas, SP, Brazil

## Abstract

**Background:**

Information is still scarce on the birthing experience of women who participate in antenatal systematic education programs. The objective of the study was to report the experience of labor as described by nulliparous women who participated and who did not in a systematic Birth Preparation Program (BPP).

**Method:**

A qualitative study was conducted with eleven women who participated in a BPP and ten women attending routine prenatal care selected through purposeful sampling. The BPP consisted of systematized antenatal group meetings structured to provide physical exercise and information on pain prevention during pregnancy, the role of the pelvic floor muscles, the physiology of labor, and pain relief techniques. A single, semi-structured interview was conducted with each participant. All interviews were recorded, transcribed verbatim and thematic analyses performed. The relevant themes were organized in the following categories of analysis: control of labor, positions adopted during labor, and satisfaction with labor.

**Results:**

Women who participated in the systematic educational activities of the BPP reported they maintained self-control during labor and used breathing exercises, exercises on the ball, massage, baths and vertical positions to control pain. Also they reported satisfaction with their birthing experience. Women who did not participate in systematic educational activities referred to difficulties in maintaining control during labor and almost half of them reported lack of control. Also they were more likely to report dissatisfaction with labor.

**Conclusions:**

Women who participated in the BPP reported self-control during labor and used non-pharmacological techniques to control pain and facilitate labor and expressed satisfaction with the birthing experience.

## Background

Educational activities aimed at preparing women for labor by providing information and practising physical exercise, breathing and relaxation techniques promote women’s self-control and may contribute for a satisfactory birthing experience. However, it has been reported that a large amount of information given over a short period of time may be ineffective; and that the lack of opportunities to discuss the information transmitted and to practise pain relief techniques may hamper the benefits of antenatal education [1,2]. There is evidence of the need to reinforce the information during labor to ensure a better effect, since the stress generated during labor and delivery may influence a woman’s memory, compromising the information received during pregnancy [3]. Also it has been argued that the inclusion of a companion, either women’s partner or somebody close to her, in pre-natal education activities to become actively involved in the use of non pharmacological techniques for labor control is beneficial for women during the birthing process [4]. However, there are still gaps on whether the information transmitted is in accordance with what pregnant women need to maintain self-control during labor [1,5].

Women’s ability to maintain self-control during labor has been seen as fundamental for a good birthing experience [6-8]. A systematic review [9] showed that women’s expectations related to self-control during delivery do not correspond to their factual experiences. Irrespective of the pain experienced, more realistic expectations and self-control during labor, seem to be directly associated with greater satisfaction. Participation in antenatal education activities was also associated with women having more realistic expectations and, consequently, more positive experiences of labor [10].

A multicenter randomized study that evaluated an antenatal educational program focusing on breathing techniques, relaxation and massage found no differences between intervention and control group with respect to the experience of the women during delivery [11]. On the other hand, another study showed that women who received guidance during pregnancy on the effects of massage or listening to music during labor reported feeling prepared for labor, having better self-control and a more positive experience of labor [12]. A Taiwan-based study reported that women who received information on exercises with a birthing ball during pregnancy and who practised these exercises during labor reported less pain and greater self-control compared to a nonintervention group [13].

There is still lack of information on the experience of women who receive guidance on non-pharmacological techniques and on the influence or benefits of systematic birth preparation programs on their well-being during labor and delivery. The objective of this study was to describe the experience of labor and delivery as reported by women who participated and women who did not participate in an antenatal program of preparation.

## Methods

A qualitative study was conducted to obtain in-depth understanding, beyond numbers, on women’s experience during labor and delivery. Qualitative research in health settings considers the perspective of the people, focuses on emotions, beliefs and values, actions and behaviors to understand the participant’s responses to health related issues, the meanings the experience has for them, and their subsequent actions. Engaging a phenomenological approach semi-structured interviews were used in the present study to understand how participants felt about their birthing experience and gain a deeper understanding of the meaning of this experience for women, to understand women’s experience of labor and delivery from their perspective and as told by them. This approach allowed the stories women told to be heard and to explore the meanings the experience had for them.

This qualitative study was conducted simultaneously with a Randomized Controlled Trial (RCT) conducted between June 2009 and September 2011 that evaluated lumbopelvic pain, urinary incontinence, anxiety levels, physical exercises, obstetric and perinatal data according to the participation or not of a Birth Preparation Program (BPP) at a public maternity teaching hospital in the southeastern region of Brazil. The study was approved by the Institutional Review Board, and informed consent was obtained from all participants prior to their inclusion in the study.

### Study sample

Primiparous women enrolled in the RCT were invited to take part in the qualitative study in the maternity ward 24 to 48 hours after delivery and before discharge. Women were approached and informed about the qualitative component of the study, they were given time to ask questions, to reflect and to decide if they wanted to participate. Those who agree were asked to sign a written consent form.

Following the logic of purposeful sampling women, from both the study and the control group of the RCT, were invited to participate in the qualitative study. The strategy used to select participants was criterion sampling [14,15]. Participants were selected according to the following criteria: primiparous women with a single full-term fetus after a low risk pregnancy, between 16 and 40 years old who had and had not participated of the BPP, who received antenatal care and delivered their babies at a public maternity teaching hospital and who had been in labor for more than 4 hours at the maternity ward without receiving any spinal anesthetics. This last criterion was established to give women the opportunity to have similar minimum time in labor without anesthetics.

Women in the intervention group of the RCT, in addition to routine antenatal care, participated in the BPP - a systemized program planned and structured specifically for the study conducted by trained physical therapists that occurred on the same days of prenatal consultation. Women participated in the BPP from 18 weeks of pregnancy onwards, on a monthly basis up to 30 weeks of pregnancy, fortnightly from 31 to 36 weeks and on a weekly basis from 37 weeks onwards. At each meeting physical exercises consisting of general stretching and strengthening exercises, pelvic floor muscle training, breathing techniques and relaxation training were performed. Also as part of the program participants received information: at 24 gestational weeks on lumbopelvic pain prevention during pregnancy and on the role of the pelvic floor muscles during pregnancy, delivery and puerperium; between 34 to 36 weeks on the physiology of labor, and non-pharmacological techniques of pain control for labor were discussed and practised; and from 37 weeks onwards doubts and necessary reinforcements were discussed and non-pharmacological techniques for pain control during labor were practised. In this program partners did not participate in the meetings.

Women of the control group (CG) of the RCT received the same routine prenatal care which included educational activities provided by the nursing staff (information on breastfeeding, the signs and symptoms of labor and visit to the maternity ward) on the same days of the consultation. Not linked to consultation days they also were invited to participate in meetings where information on physical exercise, coping techniques during labor; and information on puerperium and newborn care were provided on a free participation and non-systematic basis.

At the hospital maternity ward non-systematized guidance is given to all laboring women on non-pharmacological techniques of pain relief. A companion of women’s preference is permitted by the institution to be with the woman during labor and delivery.

### Data collection

Semi-structured interviews were conducted at the maternity unit using an interview guide (Table 1) with topics related to the experience of labor and delivery, pain control, control of labor, positions adopted during labor, and satisfaction with labor and delivery. To elaborate the interview guide, initially informal conversions, based on the objectives of the study and the topics above mentioned, were conducted with women who delivered at the same maternity ward where the study was conducted. Subsequently open-ended questions and prompts to ensure feedback from the participants on the relevant aspects of the research were organized for the interview guide.

**Table 1:** Main topics of the interview guide

1	Tell me about your experience of labor and delivery. How was it? Was it as you imagined? Was it different?
2	During labor, before you received anesthesia, was there something that helped you when you had contractions?/When you felt pain? If the answer is yes: What helped you? Can you tell more about it. Can you give some examples and explain them.
3	In your opinion were you in control during labor or not? (Control of pain, control of the contractions, of control of fear). Why?
4	What positions did you adopt during labor? Explain each one of them and tell me why you adopted those positions and about your experience with the adopted positions.
5	What is your opinion on the attitudes of the staff of the maternity ward. Were they helpful or not? Why?
6	Are you satisfied or not with your birthing experience? Why?

All interviews were recorded digitally, lasted approximately 30–40 minutes and were conducted by a physical therapist (MAM) who was involved in the elaboration and supervision of the Birth Preparation Program but did not participate in BPP activities. Therefore the interviewer had domain of the theme under discussion and no previous contact with the women interviewed. The number of interviews performed was determined by consensual agreement of the researchers that the data was meaningful for the objectives proposed for the study [14].

### Data analysis

All the interviews were transcribed verbatim and the transcripts were checked for accuracy against the recordings. Data were analyzed for thematic manifest content [14,15]. An initial thematic frame was organized based on relevant topics identified in the interviews that referred to women’s experience. During this initial phase of analysis, in the process of reading through the interviews and compiling the salient topics, the recurring ideas, experiences and behavioral patterns, similar data was organized in a meaningful way. The main themes identified and used for coding were: pain control, the positions used during labor, wellbeing, and comfort experienced in different positions, the possibility of control during labor using breathing techniques and the importance of the information received during pregnancy or in the delivery ward.

Subsequently, these themes considered relevant for the understanding of the birthing experience were organized in the following categories of analysis: control of labor, positions adopted during labor and satisfaction with labor. Data were analyzed for thematic content by one researcher (MAM) and cross-checked by another researcher (MYM).

## Results

All the women invited to take part in the study agreed to participate. Twenty-one primiparous women were interviewed after delivery and eleven of the interviewees had participated in a systematic preparation program. The women who participated in the systematic educational activities of the BPP attended a mean of six meetings. General socio-demographic and obstetric data are shown in Table 2. No substantial differences were observed in the characteristics of the women who participated in the BPP and those who did not. The following categories of analysis will be presented and discussed below: control of labor, positions adopted during labor and satisfaction with labor.

**Table 2:** Socio-demographic and obstetric data of the participants

Socio-demographic and obstetric data	Women who participated in a systematic preparation program (n = 11)	Women who did not participate in a systematic preparation program (n = 10)
Age (min – max)	18 - 29	19 - 32
Gestational age at delivery (min – max)	37 - 40	37 - 40
Education level		
Primary	6	3
High school	2	5
University	1	2
Technical college	2	0
Steady partner	9	7
Planned pregnancy	7	6
Normal delivery	6	8
Delivery without spinal or epidural anaesthesia	1	2

### Control of labor

In this category of analysis all the information identified in the interviews related to what participants believed had helped them during labor to be in control was considered. All the interviewed women associated control of labor with their ability to deal with pain and anxiety. The majority of the women who had participated in the systematic educational activities of the BPP reported that the information received helped them reduce anxiety during pregnancy and labor. Also they referred to having felt to a “sense of safety”, mainly during labor, because they had learned how to deal with pain and had resources to maintain self-control. The exception was a woman who participated in three BPP meetings who said that during labor she had been unable to remember the information received.

“*No, no control… intense pain, emotionally exhausted; I was not in control at all.” (33 years old* – CG)

*“It helped, because if it weren’t for the physiotherapists’ guidance, I certainly would not have done it as I did, with breathing…I managed to stay quite calm … it really helped.”* (20 years old – BPP)

All the women who participated in the systematic educational activities of the BPP said they had maintained control for most or all the time during labor. They reported using breathing techniques, exercises with a birthing ball, walking, massage, baths and maintaining the upright position; taking the initiative to use these techniques and that they had felt at ease using them. Also they said that the information received during the preparation became meaningful when they put the non-pharmacological techniques into practice during labor and “gained confidence” in their effectiveness as labor progressed.

*“The breathing exercises, the massages, the baths, and then, I did everything, and the positions I adopted… Because if I just stayed lying down, then the pain felt even worse; then when I sat up in that butterfly position or with my two feet together, I could put more effort into it when it contracted, and with my breathing, I could relax, and when I was able to relax, the pain was less.”* (24 years old – BPP)

*“Depending on the intensity of the pain, I breathed more shallowly or deeper; then I remembered what they said: ‘smell the flower and blow out the candle; smell the flower and blow out the candle …”* (26 years old – BPP)

The women who did not participate in systematic educational activities of preparation referred to difficulties in maintaining control during labor, and almost half of them reported lack of control. Two of the three women who had received some guidance during prenatal care on pain relief techniques said they had managed to “*have some control*” during contractions. The women who received guidance on pain control techniques during labor said that they had used one or two of these techniques, and the most frequently mentioned techniques were baths and massages. They also reported they had felt that these techniques had been insufficient to control pain.

*“I walked, I leaned on my husband, and he massaged my back. That helped, it helped a little, but only for some time, because afterwards I had no control … The pain was very intense, I could not control it, the breathing everybody was telling me to do to alleviate pain, I could not do it, the pain was intense.”* (19 years old - CG)

### Positions adopted during labor

In this category of analysis all the information on the positions women adopted during labor discussed in the interviews was considered. All the women, independently of having participated or not in the BPP, said that they had felt more comfortable during labor when they adopted an upright position. The majority considered sitting and standing up, walking around, exercising with the ball on the floor or in the shower as the most comfortable positions.

Also they said that these positions permitted pelvic mobility, were comfortable for massage, helped to relieve pain and to relax the lumbopelvic region. On the other hand these women considered horizontal positions less comfortable, since they hampered their mobility and increased the sensation of pain during contractions.

“*I think sitting was better for me…I managed to move better; I was freer to move to get at least some relief; well, a little, from the pain in my back.”* (27 years – CG)

*“The best position was under the shower, sitting under the shower with the water falling on me to alleviate the pain, but not on the bed”* (21 years old – BPP).

All of the woman who participated in a systematic preparation said that they had felt at ease to assume different upright positions and had changed positions based on how they were feeling and on what they had learned during the preparation. They said they adopted upright positions to facilitate cervical dilation and to help the progression of labor.

*“I was sitting down with the ball between my legs while holding on to that rod in the bathroom for us to support ourselves… I walked a lot along the corridor, I walked, I walked a lot.”* (29 years old – BPP)

*“I think the exercises helped a lot because it helps with the dilation too, right?”* (27 years old – BPP)

The women who did not participate in a systematic preparation reported that after receiving guidance from the staff in the labor room they began to use some upright positions during labor. Some women said that adopting vertical positions brought comfort and relieved pain, even though they were not at ease to adopt these positions or to change positions without seeking guidance from the staff.

“*They said that if I remained lying down it wouldn’t help much at delivery that it was better to walk around a bit, sit down, walk, but not stay lying down for too long.”* (20 years old – CG)

*“I went to the bathroom, there I was in the upright position, sitting on the ball and also squatting. It helped quite a lot with the pain. I felt as if it opened inside, then I separated my legs and it alleviated more it seemed as if the baby was finding its way and I felt it was better … much better.”* (23 years old – CG)

In general, both women who participated and who did not participate in a systematic preparation said that at the maternity ward there were no restrictions with regard to changing position, that the staff did not interfere when they assumed different positions and encouraged them to walk, take baths and exercise with the birthing ball.

### Satisfaction with labor

The main themes identified in the interviews that referred to satisfaction were organized in this category of analysis. The majority of the women interviewed, independently of having or not participated in the systematic preparation, said that labor had been different from what they had expected or had imagined. Some said they had imagined labor would be quicker and with less pain. On the other hand, some women said that labor was less painful than they had imagined.

*“Actually, for me, labor was something that I hadn’t expected because I thought that it was going to be something really, really slow, since I always heard stories of labor that lasted 10, 12 hours, but I was very happy, apart from the pain of course, because labor was very fast.”* (26 years old – BPP)

“*No, it wasn’t as I had imagined it. It was tiring and painful, very painful, and it took a long time before I got anesthesia and under the circumstances, actually there was no way; they couldn’t give me the anesthesia before, but because of that I was in a lot of pain and I found it very tiring”* (19 years old – CG).

All the women who participated in the BPP reported satisfaction with labor. Those who said that labor had not been long and that they had not felt much pain said they were satisfied with labor per se and because they had managed to establish control.

Those who reported intense pain were in labor for a longer time and delivered with forceps, and associated satisfaction with the birthing experience with the fact that they and their babies were well.

*“I was certainly satisfied, because I managed to maintain self-control, I felt more secure going through the delivery.”* (27 years old – BPP)

Some women who did not participate in a systematic program of preparation said they were not satisfied with their birthing experience because pain had been excessive, their husband was not present, lack of attention from the staff, insufficient physical space and the use of forceps for delivery. On the other hand, women who reported having maintained self-control during labor said they were satisfied with their labor and delivery. The reasons given for their satisfaction were: labor had not been long and pain was not “so intense”, because they received anesthesia and the care of the maternity staff was good, and mainly because they and their babies were well.

*“Well, I wasn’t very satisfied because of the pain. Because of the pain and, it was very quick and there was a moment at which I thought they weren’t paying attention to my pain”* (27 years old – CG).

*“I was very satisfied because, in the first place I thought it would be more painful when the baby was going to be born, but it did not hurt so much. I thought it would be worse than the pain of the contraction … But after I saw the baby it was over…“* (23 years old – CG)

## Discussion

In the present study, women who participated in a systemized programme of preparation for labor and delivery were satisfied with the birthing experience, managed to maintain control, and used non-pharmacological techniques for pain relief. Women who did not participate in the systemized educational activities of preparation for childbirth and who reported dissatisfaction with the experience were woman who said they had difficulties maintaining control during of labor.

The women in our study associated satisfaction with their ability to use pain control techniques to reduce discomfort during labor. These results are in agreement with previous reports [16,17] which showed that participation in a prenatal education program was associated with satisfaction with the overall childbirth experience, better and more efficient utilization of pain control techniques and feelings of control during labor. On the other hand excessive pain and lack of control were associated with a negative experience at delivery. Furthermore, results of a qualitative study showed that having control over their body during labor and the freedom to move around and change positions affected positively women’s satisfaction with the birthing experience [18].

Women in our study who participated in the systematic preparation reported that knowledge on what they had to do to control pain and how to use the non pharmacological techniques for this purpose, irrespective of the pain experienced or the duration of labor, had helped them to be in control. Previous studies have also suggested that lack of information regarding the process of labor, pain and non-pharmacological techniques for pain control are factors directly related to lack of control during labor and dissatisfaction with the birthing experience [3,10,19].

Women who participated in the BPP reported having received guidance and training on non pharmacological pain control techniques and having used these techniques during labor, a finding that is in agreement with previous results [11]. The lack of opportunities for discussion and practice of the non pharmacological pain control techniques during antenatal education makes them less effective during labor [1,2]. Knowledge acquired during antenatal preparation for labor becomes more effective when reinforced during labor [3], and this was also observed in the present study. For the BPP women the interventions reinforced and encouraged them to maintain the breathing exercises and an upright position. For the CG women the guidance and encouragement from the health professionals during labor helped them to use pain control techniques; however they reported having used fewer techniques and having less control of labor.

All the participants of this study were cared for at the same maternity ward and it can be presumed that they received similar guidance and encouragement from the healthcare staff during labor. The guidance was provided mainly by nurses, practical nurses and physiotherapists who did not have special training on non-pharmacological pain control techniques used during labor. In the majority of the Brazilian maternities there are no midwives to take on the support of laboring women since this profession does not exit in the country. Regarding the companion, women usually receive guidance that they have the right to have a companion with them during labor; however companions usually do not participate in pre-natal educational activities. It is worthwhile to reinforce that BPP did not include activities for companions.

The encouragement to adopt different upright positions during labor helped women to find comfortable upright positions during labor. A RCT previously conducted by the authors in the same hospital setting showed that the group of women who received instructions and reinforcement during labor to adopt the vertical positions spent significantly more time in the upright positions and considered upright positions more comfortable than the horizontal positions [20]. This finding was confirmed in the present study, in which the majority of the interviewed women reported they felt more comfortable in the upright rather than horizontal positions.

According to a recent review [21] although the majority of the women reported their preference for a vaginal delivery, fear of pain lead many to opt for a Caesarean section. These data evidences the importance of systematized programs to prepare women during prenatal care by providing information on labor and pain control techniques. The present study discusses the experience of labor as described by women. Women who participated in BPP activities reported having felt at ease during labor using non-pharmacological techniques for pain relief. This may give further weight to the need in settings where antenatal systematic educational activities or programs are not part of routine pre-natal care to plan and implement interventions to prepare woman for labor and delivery.

A possible limitation of the present study was the fact that spinal anesthesia is routinely used at the maternity ward where the women delivered their babies. The decision about when spinal anesthesia is performed in labor is made by the anesthetic and OBGYN staff and is not a women’s choice. Consequently it was not possible to evaluate women’s autonomy on pain relief only using the alternative of non-pharmacological coping strategies for pain control. Another possible limitation may be the fact that woman had the possibility of freely participating in routine educational interventions offered at the institution during prenatal care and for this reason most participants of the present study had taken part in some educational activity. However, some routine activities offered during pre-natal care were held outside the medical consultation days, with voluntary participation, were not attended regularly nor systematic educational intervention were performed.

A possible strength of the study lies in the fact that only primiparous women were enrolled as intent to facilitate identification of the effect of antenatal guidance on labor and delivery. A possible strength of the study was that the interviews were conducted before discharge from the maternity hospital; therefore the birthing experience was very present for these women. As a way to minimize a possible “courtesy bias”, all the interviews were conducted by a professional that did not have contact with the participants during BPP activities or other project activities.

## Conclusions

Women who received systematic guidance during pregnancy on labor and pain control techniques maintained self-control and used non-pharmacological techniques to control pain and facilitate labor and expressed satisfaction with the birthing experience. Furthermore, these results may contribute to a better understanding of the needs of women in labor and consequently help plan and organize antenatal programs to promote women’s self-control during labor and may contribute to a more satisfactory birthing experience.

## Competing interest

The authors declare that they have no competing interests.

## Authors’ contributions

MAM participated in designing the research project, was involved in the data collection, in the interpretation of the results and writing of the manuscript. MYM participated in designing the research project, interpreting the results and preparing the manuscript. JGC contributed to the final editing of the research project and manuscript and the interpretation of the results. All authors read and approved the final manuscript.

## Pre-publication history

The pre-publication history for this paper can be accessed here:

http://www.biomedcentral.com/1471-2393/13/171/prepub
